# Artificial liver support systems: what is new over the last decade?

**DOI:** 10.1186/s13613-018-0453-z

**Published:** 2018-11-15

**Authors:** Juan José García Martínez, Karim Bendjelid

**Affiliations:** 1 Intensive Care Unit, Geneva University Hospitals, 4 Rue Gabrielle-Perret-Gentil, 1205 Geneva, Switzerland; 2 Faculty of Medicine, University of Geneva, Geneva, Switzerland; 3 Geneva Hemodynamic Research Group, Geneva, Switzerland

**Keywords:** Acute liver failure, Acute on chronic liver failure, Artificial liver support, Albumin dialysis, MARS, Prometheus, SPAD, FPSA

## Abstract

The liver is a complex organ that performs vital functions of synthesis, heat production, detoxification and regulation; its failure carries a highly critical risk. At the end of the last century, some artificial liver devices began to develop with the aim of being used as supportive therapy until liver transplantation (bridge-to-transplant) or liver regeneration (bridge-to-recovery). The well-recognized devices are the Molecular Adsorbent Recirculating System™ (MARS™), the Single-Pass Albumin Dialysis system and the Fractionated Plasma Separation and Adsorption system (Prometheus™). In the following years, experimental works and early clinical applications were reported, and to date, many thousands of patients have already been treated with these devices. The ability of artificial liver support systems to replace the liver detoxification function, at least partially, has been proven, and the correction of various biochemical parameters has been demonstrated. However, the complex tasks of regulation and synthesis must be addressed through the use of bioartificial systems, which still face several developmental problems and very high production costs. Moreover, clinical data on improved survival are conflicting. This paper reviews the progress achieved and new data published on artificial liver support systems over the past decade and the prospects for these devices.

## Background

In 2016, liver diseases were responsible for more than one million deaths worldwide, and the trend has been clearly increasing in the last 10 years [1]. Some of these deaths occur in the context of liver failure, in the form of either acute liver failure (ALF) or acute on chronic liver failure (AoCLF). In ALF, the adult mortality is approximately 50%, despite the increase in the number of patients receiving liver transplants. Regarding AoCLF, some recent studies show that one-third of patients hospitalized for cirrhosis with an acute complication develop AoCLF, and their mortality thus increases dramatically [2]. In this context and given the shortage of organs for transplantation, efforts already have been developed to find therapeutic alternatives for patients who are waiting for a new organ (bridge-to-transplant) or who are not candidates for transplantation but for whom recovery is considered possible (bridge-to-recovery).

From the 1990s and onwards, several systems based on the concept of albumin dialysis have been developed, the best-known being the following: the Molecular Adsorbent Recirculating System™ (MARS™), the Single-Pass Albumin Dialysis system (SPAD) and the Fractionated Plasma Separation and Adsorption system–FPSA (Prometheus™). These systems are based on the concept of albumin dialysis and therefore on the capacity to remove the albumin-bound toxins that accumulate in liver failure. These toxins are thought to be responsible for brain failure resulting from hepatic encephalopathy (HE), renal failure due to hepatorenal syndrome, cardiovascular failure and/or an immunodepression state. These devices can also remove water-soluble substances, such as ammonia, creatinine or urea and smaller proteins such as some cytokines, by standard dialysis.

Some of the substances removed by the different artificial hepatic support systems include conjugate or unconjugated bilirubin or protoporphyrin, bile acids, glycoside derivatives, phenols, short- and medium-chain fatty acids, such as octanoate, or heterocyclic organic compounds. Removal of cytokines and other recognized inducers of HE, such as ammonia or amino acids (e.g. tryptophan or glutamine), may be a valuable tool for this major complication of liver failure [3]. Some preclinical and clinical investigations also report the removal of plasmatic nitric oxide (NO) and some pro-inflammatory and anti-inflammatory cytokines, such as tumour necrosis factor alpha (TNF-α), interleukin-6 or interleukin-10 [4, 5], even though the final balance in the setting of ALF or AoCLF and its contribution to multiorgan failure are still unknown.

In the early years following the development of the different liver devices, some clinical trials demonstrated haemodynamic and neurological benefits in their use in patients with ALF and in those with AoCLF, but many of these studies were uncontrolled and included very few patients. Some randomized controlled trials showed an improvement in survival, but the small sample size, high heterogeneity of the included patients and high variability in disease severity prevented definitive conclusions from being reached [6, 7]. However, artificial liver devices have continued to be used in many hospitals, and new experimental and clinical studies on them have been published over the past decade. In the present review, the authors emphasize new data published in this field and discuss the future of these devices.

## Methods and materials

We conducted research for relevant articles through PubMed (National Institutes of Health) and Web of Science published after 1 January 2008. The filter settings used were “English language” and “French language” and the “humans” filter. The bibliographies of the recovered articles were reviewed to identify any other relevant papers. We included randomized controlled studies preferentially, but we also discussed uncontrolled studies when the statistical comparison versus baseline was provided. We also comment on other relevant literatures.

## Molecular Adsorbent Recirculating System (MARS)

The Molecular Adsorbent Recirculating System (MARS) was originally developed by Stange et al. [8]. The technique has been available for broad clinical use since 1998. The system is composed of a blood circuit, an albumin circuit and a classic “renal” circuit. Blood is dialyzed through an albumin-impregnated high-flux dialysis membrane in such a way that hydrophobic albumin-bound toxins are released through the membrane and subsequently collected by albumin in the dialyzate. The method is based on two basic thermodynamic principles: protein-binding affinity and solute movement along a concentration gradient [9]. The elimination of toxins thus takes place through the diffusion process and depends on the free toxin concentration (which is mainly affected by the molar ratio of toxin to albumin). Toxins are cleared when passing the adsorber columns that contain activated charcoal and anion-exchange resin, and albumin is regenerated and able to accept new toxins when it passes the membrane again. Additionally, the albumin circuit itself is dialyzed in the CRRT (continuous renal replacement therapy) method, diminishing the load of water-soluble toxins.

MARS is the most widely published artificial liver support system. In the first few years following its introduction to the market, several animal and in vitro experiments and clinical studies demonstrated its capacity to remove various albumin-bound and water-soluble metabolites that accumulate in liver failure and are implicated in some of its major complications, such as HE [3, 10].

Another point of interest has been the ability of MARS to eliminate cytokines and modulate the inflammatory response involved in liver failure. Cytokines have been implicated in the development of HE, vasodilation, systemic inflammatory response syndrome (SIRS) and multiple organ failure (MOF). These proteins are believed to mediate hepatic inflammation, cholestasis and liver cell necrosis and apoptosis [11, 12]. From a theoretical standpoint, the removal of some pro-inflammatory cytokines may counteract some clinical complications of liver failure related to the inflammatory and hyperdynamic state. However, anti-inflammatory cytokines would also be removed, and the final imbalance and its contribution to multiorgan failure in the setting of ALF or AoCLF are still unknown. Old and new works show a significant elimination of some pro-inflammatory cytokines, such as TNF-α, interleukin-6 and interleukin-1 β, and anti-inflammatory cytokines, such as interleukin-10, by the MARS device [4, 5, 13]. However, other studies failed to demonstrate an effective change in the plasma cytokine concentration in patients with liver failure, perhaps due to the high rate of production in this setting [14, 15]. In 2013, Donati et al. [16] published the results of 269 MARS treatments that showed no effect on cytokine plasma levels but a significant increase in hepatic growth factor levels (a humoral hepatotrophic factor that enhances liver regeneration). Interestingly, Dominik et al. [17] demonstrated, in an in vitro study, that the removal of some cytokines could be drastically improved by using MARS with larger pore membranes, which could contribute to optimizing the cytokine plasma profile of patients. We must also consider the removal of plasmatic NO, which plays a central role in the multifactorial phenomenon of splanchnic and systemic vasodilatation and the hyperdynamic state associated with liver failure. In conclusion, the precise roles of different cytokines in the pathophysiology of liver failure and the influence of MARS on cytokine plasmatic profiles have not yet been fully elucidated over the last years. This represents undoubtedly a very interesting line of research for the future.

In recent years, some authors have been interested in other active biological substances that can also be removed by MARS. Gay et al. [18] explored the proteins dialyzed and then absorbed in the anion-exchange resin cartridge of MARS in patients with cholestasis and pruritus and found some biological relevant proteins, such as secreted Ly6/uPAR-related protein-1 (SLURP1) or defensin human neutrophil peptide–1 (HNP-1), which are involved in the inflammatory and defensive processes. In contrast, MARS does not appear to influence the plasma levels of other molecules with known immunomodulatory effects, such as neutrophil gelatinase-associated lipocalin (NGAL) or the chemokines monocyte chemoattractant protein-1 (MCP-2) and macrophage inflammatory protein-3 alpha (MIP-3α), according to some published studies [19, 20].

In vitro and in vivo studies have explored the elimination of some antibiotics by MARS, showing, for example, a significant removal of the low protein-bound antibiotics moxifloxacin and meropenem [21]. Similar results have been found with piperacillin/tazobactam. Surprisingly, one case report showed minimal removal of the highly protein-bound immunosuppressive drug tacrolimus [22]. Special attention should be paid to the dose adjustment and monitoring of some critical drugs during MARS sessions, and additional pharmacokinetic studies are required.

The optimal anticoagulation regimen during MARS has also been discussed. This is an important issue considering the difficult haemostasis balance in patients suffering from hepatic failure, who are at high risk of haemorrhagic and thrombotic phenomena. The best-known and most used method is unfractionated heparin, but there are some concerns regarding haemorrhagic risk and heparin-induced thrombocytopenia. Some studies have explored the use of continuous extracorporeal systems without anticoagulation and have found a comparable circuit lifespan [23]. In this sense, the anticoagulant-free approach may be a valid option in patients at high risk of bleeding. Local anticoagulation with citrate may also become a good option if close metabolic monitoring is exercised, and some studies have shown that it is safe and that it guarantees a longer treatment time, preventing filter loss [24, 25]. However, its widespread use cannot be recommended at this stage. In most clinical trials, unfractionated heparin was the anticoagulant of choice, but some studies have used local citrate anticoagulation, especially trials with FPSA [26, 27], with no reported adverse effects. The use of prostacyclin can be found anecdotally in the literature.

From a technical point of view, several questions have been raised about the stability of the binding properties of albumin after passing the adsorber columns or about the influence and clinical relevance of some stabilizers (such as octanoate) used in commercial albumin preparations [28, 29]. Nevertheless, there are no definitive conclusions, and these issues should be the subject of further study in the future.

With respect to clinical outcomes, several studies were published in the first years following MARS commercial availability, mostly of a retrospective and an uncontrolled nature. Most of them demonstrated benefits in terms of encephalopathy, and some showed improvement in the haemodynamic parameters. The few randomized controlled trials (RCT) assessing survival presented conflicting results [30–32]. These trials included few patients suffering from AoCLF, which was defined in a variable way according to each study. No well-conducted RCTs were published during this period in the field of ALF.

In the last decade, new studies have been performed to help understand the potential clinical benefits of using MARS. Nevertheless, patient series remain limited, definitions and inclusion criteria are strongly variable, and randomized controlled trials are scarce. We have divided recent studies found in the literature according to whether target patients suffer exclusively from ALF or AoCLF or whether both group of patients are included together (mostly in meta-analysis studies). We also report some clinical studies that point to other medical indications for MARS (miscellaneous).

### MARS for acute liver failure

Several studies on the use of MARS in the field of ALF have been published in the last decade. These studies are summarized in Table 1.

**Table 1:** Studies with clinical endpoints for ALF using MARS

Study	Years	Design	Patients number	Outcomes	Comments	LOE
Kantola et al. [35]	2008	Controlled, non-randomized MARS + SMT vs. SMT	159	No improvement in 28-day and 6-month survivals	Trend to improved survival in unknown aetiology subgroup Likely improvement in HE	2
Novelli et al. [45]	2008	Uncontrolled, prospective	6	Neurological and haemodynamic improvements*	Paediatric population	3
					PELD and SOFA improvement**	
Novelli et al. [46]	2009	Uncontrolled, retrospective	45	Number of MARS treatments associated with survival*	No improvement with MARS is a predictive factor of a fatal outcome and the need for a transplant	3
Camus et al. [47]	2009	Uncontrolled, retrospective	18	Clinical improvement	Clinical improvement compared to a control group obtained from a national register*	3
				MARS therapy associated with withdrawal from the emergency transplantation list*		
Saliba et al. [33]	2013	Controlled, randomized, multicentre MARS + SMT vs. SMT	102	No improvement in 6-month, 6-month transplantation-free and 1-year survivals	High transplant rate and short waiting time until transplant	1
					Similar adverse effects	
Lexmond et al. [36]	2015	Controlled, non-randomized MARS + SMT vs. SMT	20	No improvement in survival	Patients in MARS group were sicker	2
					Patients in MARS group received more thrombocyte transfusion*	
					Paediatric population	
Gerth et al. [37]	2017	Controlled, non-randomized MARS + SMT vs. SMT	73	No improvement in 28-day survival	Patients with graft dysfunction included	2
					No differences in outcomes between subgroups	
					Biochemical improvement**	
Hanish et al. [48]	2017	Uncontrolled, retrospective	27	Improvement in encephalopathy and in the APACHE II score**	Biochemical improvement*	3
Quintero Bernabeu et al. [49]	2018	Uncontrolled, retrospective	11	Haemodynamic improvement*	Paediatric population	3
					No significant adverse effects	

*LOE* level of evidence, determined using the strength of recommendation taxonomy (SORT) criteria [50], *SMT* standard medical therapy, *HE* hepatic encephalopathy, *PELD* paediatric end-stage liver disease, *SOFA* sequential organ failure assessment, *APACHE II* Acute Physiology and Chronic Health Evaluation II

*p < 0.01

**p < 0.05

Unfortunately, only one trial, which was presented by Saliba et al. [33], was controlled and randomized. This trial included 102 patients with ALF and without an absolute contraindication for liver transplantation. Patients were recruited from 16 liver transplantation centres across France (mostly from three centres). The trial could be rated as fair, as it scored 3 on the Oxford quality scoring system [34]. We cannot consider it to be good (scores 4–5) because the trial was not blinded. Patients received conventional treatment alone or conventional treatment combined with MARS and were stratified according to whether or not the ALF was paracetamol induced. The study failed to prove a significant difference in the overall 6-month survival and in the 6-month transplant-free survival and 1-year survival. Additionally, there was no significant improvement in encephalopathy with the MARS therapy, unlike most other published works. It is noted that the 6-month transplant-free survival was greater among the patients with paracetamol-induced ALF than that of the others (38% vs. 13%, respectively, *p* < 0.01). These disappointing results must be interpreted carefully, as the high transplantation rate and the short delay from randomization to liver transplantation in the study preclude definitive conclusions. Indeed, 14 of the 53 patients in the MARS group initially eligible for analysis were ultimately excluded because they did not receive MARS or they only received a short session of it because an organ was readily available. Sixty-six patients (64.7%) underwent transplantation, of whom 75% did so within the first 24 h following a wait-list registration. As patients with contraindication to liver transplantation were excluded from the trial, we cannot indicate whether the use of MARS might be helpful in this population and whether these patients could especially benefit from this technique (last-line treatment).

Some non-randomized controlled studies were also published, the largest being the work of Kantola et al. [35], which included 113 prospectively collected patients who received MARS and a retrospectively collected historic control group of 46 patients with whom they were compared. There was no significant difference in the 28-day and 6-month survival rates between the two groups. The native liver recovery rate of the MARS group was higher than that of the control group (49% vs. 17%, respectively, *p* < 0.01), and the transplant-free survival was also higher (66% vs. 40%, *p* < 0.05). However, the trial design and the predominantly toxic aetiology of ALF in the MARS group greatly hampered the interpretation of these results. In the most homogenous subgroup of patients with ALF of an unknown aetiology, there was a trend towards a better survival, but this was without statistical significance. The MARS patients in the other subgroups had a significantly lower model for end-stage liver disease (MELD) score compared to that of the control group, which also precludes definitive conclusions.

Two other controlled trials recently published failed to prove a survival improvement with MARS. As these are non-randomized studies, inclusion in the intervention group depended, to a large extent, on the treating physician, which is a major bias. In the work of Lexmond et al. [36], MARS treatment was reserved for the most severe patients, and these patients had a significantly higher HE grade (3.4 vs. 2, respectively, *p* < 0.01) and PELD (Paediatric End-Stage Liver Disease)/MELD score (47 vs. 38, *p* < 0.05) than those in the other group. In the study published by Gerth et al. [37], the whole cohort was composed of mildly ill patients (HE grade ≤ 1 and no vasopressor drugs). Some uncontrolled studies show haemodynamic and neurological improvements but include few patients and are of limited quality.

### MARS for acute on chronic liver failure

Trials published in the setting of AoCLF are summarized in Table 2. Only one correctly randomized controlled trial assessing survival and other clinical outcomes in this setting has been published in recent years: the RELIEF trial by Bañares et al. [38]. This relatively large multicentric trial, which scored 3 on the Oxford quality scoring system [34], failed to demonstrate a survival benefit with MARS use in both the general population included and in all of the predefined subgroups. The study showed a trend of improvement in HE in the MARS group compared to that of the control group, but this was without statistical significance. It should be pointed out that some of the exclusion criteria (platelets < 50.000/mm3, international normalized ratio > 2.3 or haemodynamic instability) could have ruled out the more severe patients. The authors also discuss the appropriateness of the schedule or dosage of the MARS sessions chosen.

**Table 2:** Studies with clinical endpoints for AoCLF using MARS

Study	Years	Design	Patients number	Outcomes	Comments	LOE
Hessel et al. [39]	2010	Controlled, randomized MARS + SMT vs. SMT	149	3-year survival improvement*	Acceptable cost-outcome ratio (measured by cost per LYG and costs per QALY)	2
					Inadequate randomization	
Novelli et al. [51]	2010	Controlled, non-randomized MARS vs. SMT	20	MELD improvement**	Delta MELD predict survival	2
Bañares et al. [38]	2013	Controlled, randomized, multicentre MARS + SMT vs. SMT	156	No improvement in 28-day and 90-day transplant-free survivals	No differences in 28-day transplant-free survival in subgroups: MELD > 20, HRS at admission, severe HE, and progressive hyperbilirubinemia	1
Gerth et al. [40]	2017	Controlled, non-randomized MARS + SMT vs. SMT	101	Improvement in 7-day** and 14-day*** transplant-free survivals	No differences in 21-day and 28-day transplant-free survivals	2
					Improvement in estimate 28-day transplant-free survival rate in subgroup of patients with two or more organs failure (CLIF-ACLF grade ≥ 2)*	

*LOE* level of evidence, determined using the strength of recommendation taxonomy (SORT) criteria [50], *SMT* standard medical therapy, *LYG* life years gained, *QALY* quality-adjusted life years, *MELD* model for end-stage liver disease, *HRS* hepatorenal syndrome, *HE* hepatic encephalopathy, *CLIF*-*ACLF* chronic liver failure-acute-on-chronic liver failure

*logrank *p* < 0.05

**p < 0.01

***p < 0.05

In a different way, a work presented by Hessel et al. [39], which was primarily designed to explore the cost-effectiveness of MARS in patients with AoCLF, showed that the cumulative survival probability at 3 years was higher in the MARS group (logrank *p* < 0.05). However, the randomization in the study was rather unclear, and the follow-up was too long to interpret the true influence of the technique on mortality.

Recently, Gerth et al. [40] published a retrospective, controlled, non-randomized study that included 101 patients with AoCLF graded according to the new Chronic Liver Failure Consortium (CLIF-C) criteria [41]. The study showed a significant reduction in early mortality in the MARS group compared to that of the standard medical therapy (SMT) group on day 7 (0% vs. 18.5%, respectively, *p* < 0.01) and day 14 (6.4% vs. 27.8%, *p* < 0.05). This effect disappeared at day 21, which could be explained by the interruption of therapy (in the MARS group, extracorporeal therapy was performed almost daily, with a median of three sessions per patient). The 14-day mortality was especially reduced among patients with two or more organ failures (9.5% in MARS group vs. 50% in SMT group, p < 0.01). Furthermore, in Kaplan–Meier estimates of the 28-day survival rate in this subgroup of patients, MARS was associated with improvement (logrank *p *< 0.05). Similarly, the authors performed a secondary analysis of the RELIEF trial data (36) using the CLIF-C criteria and showed a benefit from MARS in regard to the 14-day mortality in the subgroup of patients with two or more organ failures, but this was without statistical significance. Despite its limitations, this study suggests the necessity for further trials targeting those more severe patients suffering from AoCLF, who may especially benefit from the technique.

In addition, a meta-analysis published by Shen et al. [42] in 2016, which enrolled studies that included patients with AoCLF, showed a significative reduction in mortality with the use of artificial liver support systems. However, this meta-analysis included several non-randomized trials, and some of the studies used restrictive inclusion criteria and techniques other than MARS in the intervention group. The largest RCT used plasma exchange as liver support and included only patients with HBV-associated AoCLF [43]. These encouraging results must therefore be interpreted with caution.

Trials in the setting of AoCLF are conditioned by the absence of a worldwide recognized definition of AoCLF, which makes the selection of patients for these studies quite variable [44]. In this regard, the acceptance and use of an international definition can be a key step in optimizing future research.

### MARS for acute liver failure and acute on chronic liver failure combined

The few published trials that included ALF and AoCLF patients together are uncontrolled and retrospective. Notwithstanding, several systematic reviews and meta-analyses including RCTs published over the last 20 years have been presented recently. These studies included patients with ALF and AoCLF, and they are summarized in Table 3.

**Table 3:** Studies with clinical endpoints for ALF and AoCLF combined using MARS

Study	Years	Design	Patients number	Outcomes	Comments	LOE
Rusu et al. [59]	2009	Uncontrolled, retrospective	27	Improvement in HE in ALF**	Haemodynamic improvement in patients with liver failure post-transplantation**	3
				No clinical improvement in AoCLF		
Stutchfield et al. [54]	2011	Systematic review	8 RCT	ELS improved survival in ALF**	Independent meta-analysis for trials including patients with ALF or AoCLF	2
				No statistically significant reduction in mortality in AoCLF	3 trials using bioartificial devices included	
Vaid et al. [52]	2012	Meta-analysis	9 RCT 1 NRS	Improvement in HE*	No significant differences in subgroups (by age or MARS number sessions)	2
				No statistically significant reduction in overall mortality	Safety data no meta-analyzed	
Cisneros-Garza et al. [60]	2014	Uncontrolled, retrospective	70	Improvement in HE*	Patients with cholestasis disease were included. MARS associated with improved itching	3
Tsipotis et al. [53]	2015	Meta-analysis	10 RCT	Improvement in HE*	No significant differences in subgroups (by number of sessions or type of albumin dialysis technique)	2
				No statistically significant reduction in overall mortality	3 trials used Prometheus	
Guo-Lin He et al. [55]	2015	Systematic review	10 RCT	Reduction in mortality in ALF**	Independent meta-analysis for trials including patients with ALF or AoCLF	
				No statistically significant reduction in mortality in AoCLF	Very few patients with ALF included	2

*LOE* level of evidence, determined using the strength of recommendation taxonomy (SORT) criteria [50], *HE* hepatic encephalopathy, *RCT* randomized controlled trial, *ELS* extracorporeal liver support, *NRS* non-randomized controlled study

*p < 0.01

**p < 0.05

The meta-analysis published by Vaid et al. [52] in 2012 and the one published by Tsipotis et al. [53] in 2015 included quite similar trials. However, the work of Tsipotis included only RCT trials and was published 3 years later, allowing the authors to include the larger study by Saliba et al. [33] and complete data from the study by Bañares et al. [38], which had already been included in the meta-analysis by Vaid but which was reported as only a scientific abstract at the time. Both meta-analyses showed an improvement in hepatic encephalopathy with MARS (OR = 3.0, *p* < 0.01 in the Vaid study; RR = 1.5, *p* < 0.01 in the Tsipotis study). Disappointingly, neither meta-analysis showed a significant effect of MARS on mortality. Tsipotis also included some trials using Prometheus in its meta-analysis, such as the RCT published by Kribben et al. [26], which were meta-analyzed separately and combined with MARS trials with the same result.

Two systematic reviews, one published by Stutchfield et al. [54] in 2011 and the other by Guo-Lin He et al. [55] in 2015, conducted a separate meta-analysis for trials involving patients with ALF or AoCLF. In both studies, extracorporeal liver support significantly reduced mortality in patients with ALF compared to SMT, and no beneficial effect on survival in AoCLF was found. However, the study of Stutchfield et al. included trials using bioartificial devices, precluding any specific conclusion for MARS.

In the systematic review by Guo-Lin He et al., only trials using MARS in the intervention group were included. In the meta-analysis of the four RCTs that involved patients with ALF, the authors compared the survival in the non-transplanted patients and found that MARS therapy significantly reduced mortality compared to SMT (RR = 0.61, *p* < 0.05). This result can be confounding because very few patients were included. Furthermore, the inclusion criteria are quite different over the studies with two trials exclusively involving patients with liver failure secondary to cardiogenic shock. With the exception of the Saliba study [33], the other RCTs did not report on follow-up or allocation data, and scored low on the CONSORT score analysis [56]. One study did not have survival as a primary outcome. Nevertheless, the results are consistent across studies (*p* = 0.52; *I*^2^ = 0%) and suggest that MARS may be a valuable tool in the subgroup of patients with ALF who are critically ill.

Another meta-analysis published by Zheng et al. [57] in 2013 found a mortality benefit of artificial liver support systems over SMT in patients with liver failure. However, the study did not focus on albumin dialysis and included earlier studies using transfusion, haemoperfusion, haemofiltration and other outdated devices, such as the BioLogic-DT system.

In this regard, and in order to identify the patients who could most benefit from MARS therapy, Kantola et al. [58] analyzed 188 patients treated with MARS, most of whom suffered from ALF or AoCLF (some patients with graft failure and other liver injuries were also included). In this prospective observational study, the authors identified the aetiology of liver disease as the main factor in the survival and recovery without transplantation, with the non-transplanted patients with AoCLF having the highest mortality and probably benefitting minimally from the MARS treatment. The 1-year survival rates of all of the transplanted patients in the study were very high (91% for the ALF patients), which the authors attributed to the clinical and biochemical improvements achieved with MARS. The grade of encephalopathy prior to MARS treatment and coagulation factors levels were identified as prognostic factors in ALF.

At this time, larger RCTs with well-defined inclusion criteria are still required to confirm the benefits of MARS and to be able to widely recommend its use. In the case of ALF, which is often a life-threatening condition, the use of MARS as a bridge-to-transplant to gain time or, eventually, as a bridge-to-recovery, is warranted.

### Miscellaneous

Table 4 summarizes recently published trials in which MARS was used in clinical indications other than for ALF or AoCLF. At least one ongoing RCT was identified on the US-based clinical trials registration website (Molecular Adsorbent Recirculating System (MARS^®^) in Hypoxic Hepatitis, clinicaltrials.gov: NCT01690845). The current status of this trial is unknown.

**Table 4:** Studies using MARS in clinical indications other than for ALF or AoCLF

Study	Years	Design	Patients number	Clinical indication	Outcomes	LOE
Wong et al. [61]	2009	Uncontrolled, prospective	6	Type 1 HRS refractory to vasoconstrictor therapy	No improvement in haemodynamics	3
					No improvement in GFR; temporary improvement of creatinine during MARS**	
					Reduction in NO levels**	
Schaefer et al. [62]	2012	Uncontrolled, retrospective	3	Severe cholestatic pruritus	Paediatric patients	3
					Significant decrease in NRS score*	
					135 MARS sessions in total, during 4, 8 and 13 months prior liver transplantation	
Lavayssière et al. [63]	2013	Uncontrolled, retrospective	32	Type 1 HRS	No improvement in renal function	3
					In patients receiving norepinephrine, significant dose reductions**	
Gilg et al. [64]	2018	Uncontrolled, prospective	10	Post-hepatectomy liver failure	No improvement in MELD	3
					No major complications reported	

*LOE* level of evidence, determined using the strength of recommendation taxonomy (SORT) criteria [50], *HRS* hepatorenal syndrome, *GFR* glomerular filtration rate, *NO* nitric oxide, *NRS score* numeric rating scale, NRS 0: no pruritus, NRS 10: maximal pruritus, *MELD* model for end-stage liver disease

*p < 0.01

**p < 0.05

## Single-Pass Albumin Dialysis (SPAD)

Single-Pass Albumin Dialysis was described as an alternative to more sophisticated devices, such as MARS, in the late 1990s [65]. It is the simplest artificial liver device and can be applied in any intensive care unit employing a standard CRRT. No additional adsorbent columns or circuits are required. The patient’s blood is dialyzed through a high-flux hollow-fibre haemodiafilter using an albumin-containing dialyzate. After passing through the dialyzer, the dialyzate is discarded (in contrast to the MARS system, in which the albumin dialyzate is regenerated), and the toxins are thus removed from the system. High amounts of albumin are consumed with SPAD, making this treatment substantially expensive.

In 2004, Sauer et al. [66] published one of the first papers comparing the in vitro detoxification capacities of SPAD and MARS for water-soluble and protein-bound compounds (bilirubin and bile acids) and demonstrated that the performances of the two were similar and that both were superior to standard continuous venovenous haemodiafiltration (CVVHD). The authors used a 4.4% albumin dialyzate solution.

These results were partially confirmed in vivo by Kortgen et al. [67], who compared the detoxification capacity of the two techniques in patients with liver failure. They showed a significant decrease in the serum bilirubin level with both treatments. However, only with MARS did the creatinine and urea levels significantly decrease. There were no significant differences in the other biochemical, haemodynamic or clinical values. The study was retrospective and non-randomized, and there were far fewer patients in the SPAD arm than there were in the MARS arm. The authors performed SPAD with a 5% albumin dialyzate solution and a low dialyzate flow rate (700 mL/h), which probably influenced the results.

The determination of the optimal albumin concentration in the dialyzate solution or that of the most efficient dialyzate flow rate when carrying out SPAD was already addressed by Churchwell et al. [68] in 2009. They compared the effect of various blood flow rates, dialyzate flow rates, dialyzate albumin concentrations (0%, 2.5% and 5%) and dialyzers on the clearance of some highly protein-bound drugs (valproic acid and carbamazepine). The authors demonstrated that the highest extraction ratios were achieved using the combination of 5% albumin dialyzate and a larger polysulfone dialyzer (surface area 1.5 m^2^). Two years later, Benyoub et al. [69] showed significant reductions in the bilirubin and bile acid levels in patients suffering from ALF or AoCLF from using SPAD with a 3.2% albumin dialyzate and a 1000 mL/h dialyzate flow rate. More recently, Schmuck et al. [70] found, in an in vitro model, an optimal detoxification efficiency for albumin-bound substances (bilirubin and bile acids) with a 3% albumin concentration and a dialyzate flow rate of 1000 mL/h. They used SPAD with conventional CVVHD and a high-flux polysulfone haemodiafilter.

With respect to clinical data on SPAD, only a few case reports were published in the early years, and there are currently no published studies that focus on demonstrating the clinical benefits of SPAD versus standard medical therapy (SMT) in ALF or AoCLF. Two retrospective uncontrolled studies reviewing data from patients with liver failure treated with SPAD as rescue therapy were identified. One included paediatric patients with ALF of different aetiologies [71], and the other included adults patients with severe liver dysfunction in a context of alcoholic liver disease who were treated with SPAD or Prometheus [72]. Neither of these studies allow us to draw conclusions about the clinical usefulness of SPAD, and they only show us its relative ease of use and the absence of unexpected complications from its use.

The only randomized study using SPAD was recently published by Sponholz et al. [73]. This is a randomized, controlled crossover study comparing the detoxification capacity and influence on clinical and paraclinical parameters of SPAD (4% albumin dialyzate solution; 700 mL/h dialysis flow rate) and MARS (20% albumin flow rate equal to the blood flow rate, 2000 mL/h dialysis flow rate). The authors found similar reductions in the total plasma bilirubin levels, without significant differences between the two devices. The reductions in the total bile acids and γ-glutamyl transferase levels in the SPAD arm were non-significant. The creatinine and urea levels were not significantly reduced with SPAD compared to those of MARS. In contrast to other studies, neither MARS nor SPAD induced a reduction in the systemic cytokine levels. Moreover, the patients treated with SPAD presented some metabolic derangements such as increasing lactate levels or decreasing calcium levels, which are probably explained by the preferential use of citrate anti-coagulation with a low dialysis flow rate. The effects of MARS and SPAD on the clinical parameters (HE and haemodynamic status) were small and equivalent.

Currently, SPAD may be an easy-to-use alternative to MARS, but the optimal albumin dialyzate concentration, dialyzate flow rate and treatment regimen are not yet fully established. A new randomized crossover trial comparing MARS and SPAD (with the change in the plasma levels of total bilirubin as the primary endpoint, and with tolerance, change in bile acid levels, change in conjugate bilirubin levels, pulsatility index of the middle cerebral artery and HE score as the secondary endpoints) is underway. The recruitment phase of the study has actually completed (clinicaltrials.gov: NCT02310542).

## Fractionated Plasma Separation and Adsorption–FPSA (Prometheus)

The FPSA method was first described by Falkenhagen et al. [74]. While MARS uses an exogenous albumin solution, the available Prometheus machine allows for the patient’s own albumin to enter the first circuit using the AlbuFlow^®^ filter (molecular cut-off of 250 kDa). Albumin is reactivated and returned to circulation using a neutral resin adsorber (Prometh^®^ 01) and an anion-exchange column (Prometh^®^ 02). The blood then enters a second circuit where it is treated by conventional high-flux haemodialysis before being returned to the patient.

In the first 10 years after its appearance on the market, some studies demonstrated the in vitro and in vivo efficacy of Prometheus in clearing ammonia, bilirubin or bile acids, showing that it performs even better than MARS does [75].

These results have been confirmed in recent years. In 2009, Grodzicki el al. [76] showed significant decreases in serum ammonia, bilirubin, aspartate aminotransferase, alanine aminotransferase, urea and creatinine with the use of FPSA in patients suffering from ALF. Rifai et al. [77] demonstrated a decrease in almost all twenty-six of the amino acids measured in nine patients with liver failure (eight of them with an HE grade of 2 or more) with a single FPSA session. Some of the amino acids cleared (such as glutamine, phenylalanine, tyrosine and tryptophan) have been directly implicated in the development of HE, suggesting that FPSA may improve this serious condition associated with liver failure. In another study involving patients with ALF monitored by cerebral microdialysis, the authors found the same trend in the removal of aromatic amino acids (especially phenylalanine) from the arterial blood after a single FPSA session, but it was surprisingly without a significant change in the concentrations measured in the microdialyzate [78]. It should be noted, in this regard, that one of the inclusion criteria was a high risk of intracranial hypertension, but the patients did not need to have clinical encephalopathy. On the same topic, we found an interesting paper published by Ryska et al. [79] that described the influence of Prometheus therapy on the evolution of intracranial pressure (ICP) in a well-conducted experimental model of ALF in pigs. The authors showed a significant reduction in the ICP in the group treated with FPSA compared to that of the control group (24 mmHg vs. 29.8 mmHg, respectively, 12 h after liver devascularization, *p *< 0.05) again indicating the probable usefulness of Prometheus in HE.

On the removal of cytokines and other molecules involved in the development and evolution of liver failure, Rocen et al. [80] measured the concentrations of cytokines, inflammatory markers (C-reactive protein and procalcitonin) and liver regeneration markers, such as hepatocyte growth factor (HGF) and α1 fetoprotein, during FPSA sessions in eleven patients with ALF of different aetiologies (nine of whom were finally transplanted). The authors showed a significant decrease in most cytokines and inflammatory marker concentrations with Prometheus, which contrasts the results of previous research [81]. Nevertheless, no clinical improvement, except for improvement of encephalopathy, was demonstrated. Surprisingly, the HGF concentration increased significantly, which could favour hepatic regeneration. As with MARS, the ability of Prometheus to influence the cytokine profile and the final clinical outcome of this action are still unclear and require further research.

Few clinical studies evaluating the survival or other clinical outcomes with Prometheus were published in the 2000s. A randomized controlled trial published during this period by Laleman et al. [81] compared SMT with MARS and Prometheus in patients presenting AoCLF, and only MARS showed benefits in some of the included haemodynamic variables, such as mean arterial pressure, stroke volume or systemic resistances. Survival was not assessed in this study. Dethloff et al. [82] showed the same tendency to improve mean arterial pressure only with MARS treatment in a randomized controlled study comparing MARS, Prometheus and conventional haemodialysis in patients with decompensated cirrhosis. These different actions on haemodynamics of MARS and Prometheus have no obvious explanation. As described above, MARS and Prometheus may influence cytokine and inflammatory molecules concentrations, thus inducing haemodynamic changes, but the final balance for both is unknown. Several studies have demonstrated a reduction in NO levels with MARS [4, 81], which is probably removed in its main circulating complexed form, S-nitroso-serum albumin [83], but the use of Prometheus probably also influences NO levels [84]. In addition, most of the clinical trials published so far using MARS or Prometheus in AoCLF did not use haemodynamic outcomes and description of haemodynamic changes with treatment was often not included. It should also be noted that haemodynamically unstable patients were systematically excluded in these studies.

Over the last few years, only a limited number of studies have used clinical endpoints (Table 5). The most important was the HELIOS study, which was published in 2012 by Kribben et al. [26]. This is a multicentric randomized controlled trial comparing Prometheus with SMT in 145 patients with AoCLF, and the primary endpoint was the probabilities of survival at 28 days and 90 days (irrespective of liver transplantation). This RCT scored 3 on the Oxford quality scoring system. This trial failed to prove a survival benefit with Prometheus in the overall patient population, and the patient recruitment was interrupted after the interim analysis (90 patients) due to futility (204 patients were initially planned for inclusion in the study). It is important to note that in the overall population the probability of survival was slightly higher in the Prometheus group compared to the SMT group (90-day survival probability: 47% vs. 38%) but without statistical significance.

**Table 5:** Studies with clinical endpoints using Prometheus

Study	Years	Design	Patients number	Liver disease	Outcomes	LOE
Sentürk et al. [27]	2010	Uncontrolled, prospective	27	ALF AoCLF	Biochemical improvement Improvement in HE*	3
Kribben et al. [26]	2012	Randomized, controlled, multicentric Prometheus + SMT vs SMT	145	AoCLF	No improvement in 28-day and 90-day survivals, except in subgroup with MELD > 30 Similar adverse effects	1
Bergis et al. [86]	2012	Controlled, non-randomized, multicentric	20	Amanitas phalloides intoxication and liver dysfunction	No statistically significance difference in survivals	2
Komardina et al. [85]	2017	Uncontrolled, prospective	39	Ischaemic ALF	Haemodynamic and biochemical improvements**	3

*LOE* level of evidence, determined using the strength of recommendation taxonomy (SORT) criteria [50], *HE* hepatic encephalopathy, *SMT* standard medical therapy, *MLED* model for end-stage liver disease

*p < 0.05

**p < 0.01

Among the predefined subgroups analyzed, the survival probability of patients with more severe liver disease (MELD > 30) treated with Prometheus was significantly higher than that of the patients treated with SMT alone (90-day survival probability: 48% vs. 9%, respectively, logrank *p* < 0.05). In the subgroups of patients with less sever disease (MELD < 20 and MELD 20–30), differences in survival are not statistically significant. This may indicate the usefulness of Prometheus when applied to more severe patients, but this conclusion is strongly limited by the small size of the subgroups.

Some other studies, although quite heterogeneous, have been published in recent years. Sentürk el al. [27] compared the biochemical and clinical parameters during FPSA in patients with ALF and AoCLF, demonstrating a significant improvement in the biochemical parameters and in HE. Survival was not assessed in this study. Komardina et al. [85] described haemodynamic and biochemical improvements with Prometheus in patients with ischaemic ALF (complication after cardiac surgery), but no change in the survival was shown. Surprisingly, the overall mortality was very high in the study (only 9 of the 39 patients included were alive at day 28).

## Other devices

Some new artificial devices, or modifications to currently used systems, have been developed in the past few years.

Marangoni et al. [87] presented the so-called high-efficiency MARS by inserting a double adsorption unit (double columns containing charcoal and another pair with ion-exchange resin, mounted in parallel) into the albumin circuit. The authors compared the detoxification capacity of their system with that of the “classical” MARS in four patients with liver failure and showed that the “improved” MARS was notably more effective in removing bilirubin and bile acids.

An hybrid extracorporeal protocol was presented by Akcan Arikan et al. [88], which combined high-flux CRRT for hyperammonaemia, therapeutic plasma exchange for coagulopathy and MARS for hepatic encephalopathy. They presented this protocol in a retrospective observational study that included fifteen paediatric patients with ALF or AoCLF, who were supported with this therapy and showed improvement in HE.

In 2017, one experimental study by Al-Chalabi et al. [89] using an animal model of ALF in pigs and one uncontrolled clinical trial by Huber et al. [90] including 14 patients with liver failure were published, both of which used a new device called ADVOS (ADVanced Organ Support). The laboratory prototype of ADVOS was first presented in 2013 [91] and included an extracorporeal blood circuit, a dialyzate circuit containing standard dialyzate with a 2–4% albumin concentration and a third circuit where the albumin dialyzate was divided into two parts. Before reaching the cation and anion filters, each part undergoes a change in the pH value by the addition of acid or base and is subjected to a temperature change, resulting in a release of albumin-bound toxins. The resulting dialyzates containing toxin-free albumin join with each other in order to reach the desired pH before entering the haemodialyzer again. In the animal study mentioned above, all of the animals treated by ADVOS survived (5 out of 10), whereas the pigs treated with SMT died in the 10-h observation period (*p* < 0.01). Significant haemodynamic and biochemical improvements were demonstrated with ADVOS. A significant decrease in the bilirubin level was also demonstrated in the clinical trial published by Huber et al. [90]. No further clinical studies on this promising technique have been published so far.

Some modifications of the techniques described above, such as plasma diafiltration and some protocols already used several years ago to treat liver failure, such as plasma exchange or therapeutic apheresis using a bilirubin adsorbent column, are also found anecdotally in the literature [43, 92, 93].

## Conclusion

Severe liver failure is associated with high mortality, as many patients die despite undergoing optimal medical treatment. Even if liver transplantation has emerged as an essential therapy, many patients with this disease will unfortunately die while waiting for a hepatic transplant. Consequently, there is a clear need for a liver support system to provide a “bridge” to a final treatment. Over the last two decades, several artificial liver support systems with promised advances were introduced. However, whether such improvements could be translated into survival benefit is still uncertain, given the scarcity of available results of RCTs. The present reality is probably related to several factors, including the involvement of several interconnected organs and the fact that liver failure patients constitute a heterogeneous population with severe multimorbidity [94]. Moreover, there is no precise recommendation on the effective timing of the initiation of artificial liver support systems. In this regard, the future prospects of artificial liver support systems should rely on the completion of adequately powered RCTs addressing these crucial clinical issues and endpoints. New indications for this organ support, such as post-hepatectomy liver failure, should also be explored. In the meantime, and in the absence of alternative options to support this vital organ, it is difficult to criticize the cautious use of these secured artificial liver devices as “salvage” therapy in patients suffering from ALF or severe AoCLF.
